# Genetic Mapping and Discovery of the Candidate Gene for Black Seed Coat Color in Watermelon (*Citrullus lanatus*)

**DOI:** 10.3389/fpls.2019.01689

**Published:** 2020-01-22

**Authors:** Bingbing Li, Xuqiang Lu, Haileslassie Gebremeskel, Shengjie Zhao, Nan He, Pingli Yuan, Chengsheng Gong, Umer Mohammed, Wenge Liu

**Affiliations:** Zhengzhou Fruit Research Institute, Chinese Academy of Agricultural Sciences, Zhengzhou, Henan, China

**Keywords:** watermelon, seed coat color, polyphenol oxidase, genetic mapping, candidate gene

## Abstract

Seed coat color is an important trait highly affecting the seed quality and flesh appearance of watermelon (*Citrullus lanatus*). However, the molecular regulation mechanism of seed coat color in watermelon is still unclear. In the present study, genetic analysis was performed by evaluating F_1_, F_2_ and BC_1_ populations derived from two parental lines (9904 with light yellow seeds and Handel with black seeds), suggesting that a single dominant gene controls the black seed coat. The initial mapping result revealed a region of interest spanning 370 kb on chromosome 3. Genetic mapping with CAPS and SNP markers narrowed down the candidate region to 70.2 kb. Sequence alignment of the three putative genes in the candidate region suggested that there was a single-nucleotide insertion in the coding region of *Cla019481* in 9904, resulting in a frameshift mutation and premature stop codon. The results indicated that *Cla019481* named *ClCS1* was the candidate gene for black seed coat color in watermelon. In addition, gene annotation revealed that *Cla019481* encoded a polyphenol oxidase (PPO), which involved in the oxidation step of the melanin biosynthesis. This research finding will facilitate maker-assisted selection in watermelon and provide evidence for the study of black seed coat coloration in plants.

## Introduction

Watermelon [*Citrullus lantus* (Thunb.) Matsum. & Nakai (2n = 2x = 22)] is an important horticultural crop worldwide (Mohr, 1986; Levi et al., 2001). Seed coat color is an essential part of botanical character in watermelon which plays an important role in watermelon breeding, especially for seeded watermelon (Mavi, 2010). However, there are few molecular studies for seed coat color compared to other traits (Yan et al., 1996; Łopusiewicz, 2018). Seed coat color is also associated with the biochemical characteristics of seeds, the amounts and activities of antioxidants (El-Bramawy et al., 2008), and affects the flesh appearance (Wehner, 2008). Black seed is attractive matched with red canary yellow flesh, while white or light seed color is an ideal character for the near-seedless cultivars (Wehner, 2008).

Seed coat has a large diversity of colors in different species, genotypes and even at different seasons or developmental stages (Demir et al., 2004; Wan et al., 2016). The mechanism of seed coat coloration has been well-illuminated in Brassicaceae family, especially in the model species of *Arabidopsis* and its close phylogenetic relative *Brassica* (Debeaujon et al., 2000; Baxter et al., 2005; Li et al., 2010; Yu, 2013; Padmaja et al., 2014). Seed coat color in *Arabidopsis* and *Brassica* is generally categorized into two main classes, yellow and brown (Yu, 2013). The yellow seed is formed due to the transparent and colorless seed coat, resulting in the exposure of yellow embryo (Sagasser et al., 2002; Bharti and Khurana, 2003). Flavonoids (flavonols and proanthocyanidin) are the main components for the seed coat coloration in *Arabidopsis* and *Brassica*. The endothelium layers synthesize proanthocyanidins (PAs) that condense into tannins and oxidize, resulting in a brown color in mature seeds (Yu, 2013).

In recent years, dozens of genes associated with flavonoid biosynthesis have been well characterized by TRANSPARENT TESTA (*tt*) mutants (Debeaujon et al., 2000; Baxter et al., 2005; Li et al., 2010; Yu, 2013; Padmaja et al., 2014). Nearly 27 mutations have been detected to show association with the flavonoid pathway related to the seed coat color in *Arabidopsis* (Yu, 2013). Several genes identified at the molecular level have been categorized into two groups of structural proteins: the early biosynthetic genes (EBGs) including chalcone synthase (*CHS*), chalcone isomerase (*CHI*), flavanone-3-hydroxylase (*F3H*), and flavanone-3’-hydroxylase (*F3*’*H*); the late biosynthetic genes (LBGs) including dihydroﬂavonol reductase (DFR), leucocyanidin dioxygenase (LDOX) and anthocyanidin reductase (ANR). The expression of the underlying biosynthetic genes was regulated by some regulatory factors (*TT1*, *TT2*, *TT8*, *TT16*, *TTG1*, *TTG2*, *PAP1*, *GL3*, *ANL2*, *FUSCA3*, *KAN4*) (Qu et al., 2013; Yu, 2013; Padmaja et al., 2014). In addition to flavonoid, it was reported that melanin also affected the seed coat color of seeded rapes (Zhang et al., 2006). The seed coat color mainly depends on the content of melanin in the last stage of seed development (Zhang et al., 2006; Yu, 2013). Melanin is a widespread black compound in nature, especially in the seed coat (Park and Hoshino, 2012; Wan et al., 2016). Natural melanin has potential values for pharmacology, cosmetics and functional foods due to its antioxidant, radio-protective, antitumor, antiviral, antimicrobial and anti-inflammatory properties (Łopusiewicz, 2018). However, there are limited studies about the candidate genes regulating melanin biosynthesis controlling the seed coat coloration, and the genetic regulatory mechanisms accounting for black seed coat coloration has not been well elucidated (Yu, 2013).

Polyphenol oxidase (PPO) is the key enzyme catalyzing phenolic compounds to form o-quinones, which could easily react with amines, proteins or other phenols to produce dark melanin pigments (Mayer and Harel, 1979; Matheis and Whitaker, 1984; Whitaker and Lee, 1995; Yu, 2013). In higher plants, it has been reported that PPO is responsible for the dark color of damaged kernels, fruits, or vegetables, which may be involved in disease resistance (Yu et al., 2008). *SiPPO* encoding polyphenol oxidase was proposed to be responsible for generating black or whiter sesame (Wei et al., 2015).

In watermelon, previous studies for seed coat color mainly focused on the inheritance, antioxidant of the seed coat color and the extraction of pigment. Watermelon exhibits a wide range of seed coat colors, commonly white, tan, brown, black, red, green, and dotted (Poole, 1941). McKay (1936) suggested that three genes determined watermelon seed coat color: *r*, *w*, and *t* for red, white, and tan, respectively. The interaction of the three genes produced six patterns: black *(RR TT WW*), clump (*RR TT ww*), tan (*RR tt WW*), white with tan tip (*RR tt ww*), red *(rr tt WW*), and white with pink tip *(rr tt ww*) (Kanda, 1931; McKay, 1936; Poole, 1941). A modifier, *d* was suggested to produce a black and dotted seed coat (Poole, 1941). Paudel et al. (2019) developed three segregating F_2_ populations to re-investigate the four-gene model and map the locus of the four genes. Paudel et al. (2019) found that the inheritance of the *T* locus did not fit the four-gene model in F_2_ progenies (dotted black × red). The tannish seed coat color was observed and classified as tan^1^ that was affected by *T* ^1^, a novel locus or a different allele of the *T* locus (Poole, 1941; Paudel et al., 2019). Besides, the *R*, *T*, *W*, and *D* loci were mapped on chromosomes 3, 5, 6 and 8, respectively. In watermelon, melanin accumulation in seed coat was responsible for the black seed coloration and black seeds were considered as a potential source of natural melanin (Yan et al., 1996; Łopusiewicz, 2018).

Until now, there are no concrete molecular evidences in watermelon seed coat coloration. Studies on the melanin pigmentation in plant seed coloration are not plentiful. In this study, we aimed to illustrate the inheritance of watermelon seed coat color and detect the candidate gene responsible for black seed coat coloration. The present study will facilitate marker-assisted selection and provide additional evidence for the melanin pigmentation in plant seed coat.

## Materials and Methods

### Plant Materials and Genetic Mapping Population

The preliminary mapping population consisted of 126 recombinant inbred lines (RILs, F_7_), derived from a cross between the inbred lines 9904 (female parent) and Handel (male parent) which have light yellow and black seed coat, respectively (Li et al., 2018). In the segregating population, there was a third phenotype of seed coat color named dotted, which was not completely black (black strip or dot) seed coat (Poole, 1941; Li et al., 2018). The F_2_ population was used to perform fine mapping. The backcross population was produced by hybridizing F_1_ plant with each parent to create BC_1_P_1_ (F_1_×9904) and BC_1_P_2_ (F_1_×Handel) and used to validate genetic inheritance of seed coat color.

For the genetic map construction and segregation analysis, the RIL population was grown together with the parental lines at two locations under three environments: Sanya Experimental Station in 2016 (Hainan, open field) and Xinxiang Experimental Station in 2017 (Henan, greenhouse and open field) (Li et al., 2018). The F_2_ population was grown in 2018 (Henan) during spring season with 560 individuals. The 140 BC_1_P_1_ and 161 BC_1_P_2_ individuals were grown in 2018 autumn season (**Table S1**). The phenotype of seed coat color was determined by visual observation, and watermelon seeds were categorized into black, light yellow and dotted groups based on their appearance at 40 days after pollination (DAP).

### Measurement of Melanin, Polyphenol and Flavonoid Content

The mature seed coat of the parental lines at 40 DAP were collected and pigments (melanin, polyphenol and flavonoid) were extracted according to the methods described by Ye et al. (2001a) and Li et al. (2011). Three biological and technical replicates were used for the measurement. Average content for each sample was calculated.

### Determination of Polyphenol Oxidase Activity

Seed coat samples (18 and 26 DAP) without the cotyledon and embryo were collected from parental lines by scalpels, and then were used to measure the activity of PPO. PPO was extracted and measured using the PPO Assay Kit (Solarbio, China) according to the manufacturer’s instruction.

### DNA Extraction, Analysis of Whole-Genome Resequencing Data, and Genetic Map Construction

Young leaves from two parental lines and the segregating population were collected and stored at −80°C until DNA was extracted. The genomic DNA was extracted by the cetyltrimethyl ammonium bromide (CTAB) method (Murray and Thompson, 1980). DNA was quantified with a NanDrop-1000 spectrophotometer (NanoDrop, USA) and was evaluated by electrophoresis in 1.0% agarose gel.

In our previous study (Li et al., 2018), we constructed a high-density genetic map based on whole-genome resequencing of the RIL population and both parental lines. A total of 7.67 Gbp, 8.81 Gbp, and 177.08 Gbp of high-quality reads were obtained from the 9904, Handel, and RIL population, respectively. The average coverage depths of the markers were 19-fold for the male parent, 17-fold for the female parent, and 3-fold for the RIL population. The average Q30 ratio was more than 85%, and the average GC content was nearly 35% for the RIL individuals (Li et al., 2018). The distribution of SNP mutations and coverage of assembly scaffolds by high-quality reads indicated that the genome resequencing was sufficiently random (Li et al., 2018). A total of 178,762 SNPs with at least a 4-fold sequencing depth were obtained by analyzing the parental lines. All of the SNP sites in the RILs were integrated into a recombination bin unit, and 2,132 recombinant bins comprising 103,029 SNPs were used to construct the genetic map (Li et al., 2018). As compared to other recent studies in watermelon, we found that greater number of SNP markers were mapped to this genetic map (Li et al., 2018). The final high-density genetic map had a total length of 1,508.94 cM, with an average distance of 0.74 cM between adjacent bin markers. Additionally, the haplotype, heat maps and collinearity of the genetic map with watermelon reference genome showed that the high-density genetic map was accurately assembled with good quality (Li et al., 2018). The LOD thresholds for determining significant loci were estimated from 1,000 permutations and a minimum LOD score of 2.5 was used to judge the presence of loci on the chromosome (Churchill and Doerge, 1994).

### Molecular Marker Development and Genetic Mapping

Re-sequenced data were compared with the available ‘97103’ watermelon reference genome version 1 from the Cucurbit Genomics Database (http://cucurbitgenomics.org/) to identify reliable SNPs through a filter pipeline (Takagi et al., 2013). To narrow down the candidate region and verify the accuracy of the preliminary mapping derived from the genetic map, the corresponding cleaved amplified polymorphic sequence (CAPS) markers were developed based on SNPs (**Table S2**). Finally, 57 CAPS makers were developed to screen F_2_ population (540 individuals) for fine mapping (**Table S2**).

PCR amplification was performed in a 10 µl reaction with 1 µl DNA, 5 µl PCR master mix, 0.5 µl of 10 µM per primer, and 3 µl distilled water. The PCR protocol was performed under the following conditions: initial denaturation at 94°C for 1 min and 30 s; followed by 30 cycles at 94°C for 20 s, 57°C for 20 s, 72°C for 50 s; and a final extension at 72°C for 5 min. Then, the corresponding restriction endonucleases were used to digest the amplified PCR products at 37°C or 65°C for 4–10 h following the manufacturer’s instructions. The digested products were separated on 1.0% agarose gels and visualized with a Versa Doc (Bio-Rad). The markers with polymorphisms were used for fine mapping.

### RNA Isolation and Quantitative Real-Time PCR Analysis of the Candidate Gene

The seed coat samples from different developmental stages (18 and 26 DAP) and other tissue samples, including roots, stems, leaves, and male flowers were collected from both parental lines. RNA was isolated using the plant total RNA purification kit (TIANGEN, China) according to the manufacturer’s instructions and then the first-strand cDNA was synthesized using a cDNA synthesis kit (Takara, Japan).

The gene-specific primers of the candidate genes and reference gene *Actin* (Kong et al., 2015) for quantitative real-time PCR (qRT-PCR) were designed based on the Cucurbit Genomic Database (http://cucurbitgenomics.org), using the software Primer Premier 5. The expression levels of the candidate genes were performed using a LightCycler480 RT-PCR system (Roche, Swiss) with a Real Master Mix (SYBR Green) kit (Toyobo, Japan). Amplification was carried out in a 20 µl reaction mixture containing 1 µl cDNA, 1 µl forward and reverse primers (10 µM), 10 µl 2 × SYBR Green real-time PCR mixes, with nuclease-free water added to a total reaction of 20 µl. Three biological and technical replicates were used for qRT-PCR. Average relative expression levels for each sample were calculated. The expression level was analyzed by the 2^−△△Ct^ method (Livak and Schmittgen, 2001), and the primer sequences used in this study are listed in **Table S3**.

### Sequence and Phylogenetic Analysis of the Candidate Gene

The sequence and gene function were retrieved from the Cucurbit Genomics Database (http://cucurbitgenomics.org). DNA and amino acid sequences were aligned using DNAMAN (version 9). Phylogenetic analysis were performed using MEGA 7 software with a bootstrap method and 1000 replications (Kumar et al., 2016).

## Results

### Inheritance and Phenotypic Characterization of Seed Coat Color in Watermelon

In the present study, the parental lines showed significant variations in seed coat color. 9904 has light yellow seed coat, while Handel has black seed coat color. In F_1_ population, all the seeds were black without segregation, which revealed that black was dominant to light yellow seed coat color. There was a third phenotype of seed coat color named dotted, which was not completely black (black strip or dotted) seed coat in the F_2_ population (Poole, 1941; Li et al., 2018). The F_2_ population separated into 332 plants with black seed coat, 101 plants with dotted seed coat and 127 plants with light yellow seed coat, resulting in good fit to a 9:3:4 segregation ratio (χ^2^ = 2.28, P = 0.32) (**Table S1**). For the BC_1_P_1_ population, there were 40 plants with black seed coat, 32 plants with dotted seed coat and 68 plants with light yellow seed coat, showing a ratio of 1:1:2 (χ^2^ = 1.03, P = 0.60). While all the 161 individuals of BC_1_P_2_ had black seed coat. When seeds from the F_2_ and BC_1_ generation were scored as black (black and dotted) vs nonblack (light yellow), the seed phenotype fits a typical Mendelian segregation ratio of 3:1 (χ^2^ = 1.61, P = 0.2) and 1:1 (χ^2^ = 0.11, P = 0.73), respectively. According to the genetic analysis, we detected that two genes account for the seed coat color in present materials resulting in three different phenotypes, black (*W D)*, dotted (*W d d*), and light yellow (*w w)*. The *W* gene was responsible for the black coloration, while *d* accounted for the distribution of the black in seed coat resulting in partial black named dotted. Besides, when seeds were scored as black (black and dotted) and nonblack (light yellow), the phenotype of the seed coat perfectly fit a ratio of Mendelian single gene segregation ratio, black (*W*): light yellow (*w w*), which indicated that black seed coat is controlled by a single dominant gene (*W*).

### Genetic Mapping of the Candidate Gene

**Figure 1:**
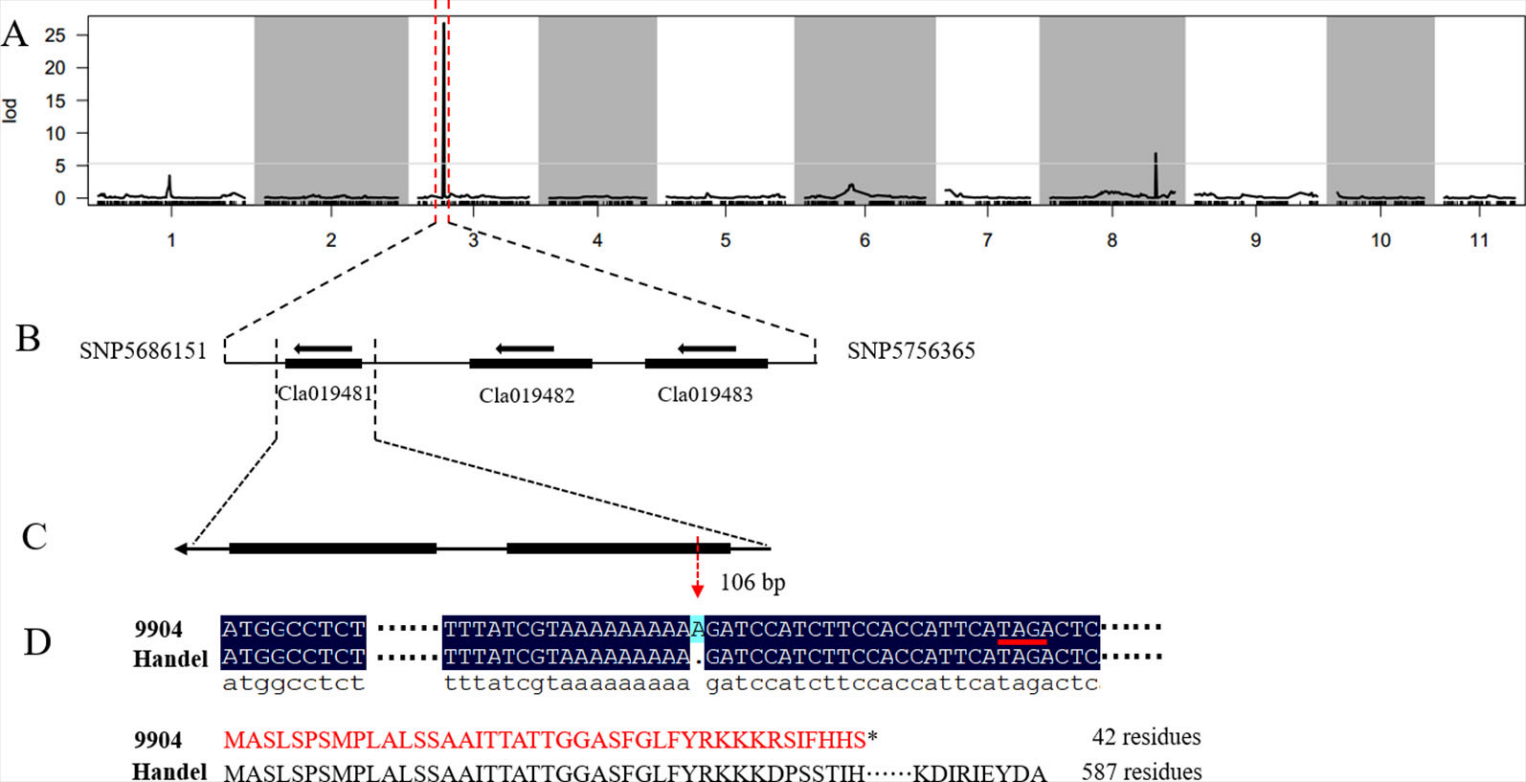
Genetic mapping of the seed coat color gene and candidate gene analysis in watermelon. **(A)** The candidate gene was located on chromosome 3. **(B)** The candidate gene was localized to a 70.2 kb region between the flanking markers SNP5686151 and SNP5756365. **(C)** Structure of the *Cla019481* gene. **(D)** A frameshift mutation and early termination of translation in 9904.

In our previous study, we detected a prominent locus (qsc-c3-1) associated with black seed coat color (Li et al., 2018). To narrow down the genetic region and identify the candidate genes for watermelon seed coat color, the ampliative F_2_ population with 560 individuals was developed. Based on the ‘97103’ watermelon reference genome version 1 (http://cucurbitgenomics.org), 57 CAPS and 10 SNP markers were developed in the candidate region on chromosome 3 to screen all F_2_ individuals for polymorphic analysis (**Table S2**). Finally, the candidate gene was delimited to a 70.2 kb region between SNP5686151 and SNP5756365 with five recombinant individuals (**Figure 1**, **Table 1**). The candidate gene responsible for black seed coat color was delimited in to a nearly 70.2 kb interval on chromosome 3 (**Figure 1**).

**Table 1:** Phenotypes and genotypes of recombinant individuals showing the recombinant breaking points.

No.	Phenotype	Chr3_5654223	SNP5666004	SNP5686151	SNP5707786	SNP5733877	SNP5756365	SNP5822743	SNP5832388	SNP5848889
1	LY	a	a	a	a	a	a	a	a	a
2	LY	a	a	a	a	a	a	a	a	a
3	LY	H	a	a	a	a	a	a	a	a
4	B	H	H	H	H	H	H	H	H	H
5	B	a	a	a	A	H	H	H	H	H
6	LY	H	a	a	a	a	a	a	a	a
7	B	a	a	a	A	H	H	H	H	H
8	B	H	H	H	H	H	a	a	a	a
9	B	a	A	A	A	A	A	A	A	A
10	B	a	A	A	A	A	A	A	A	A
11	B	A	A	A	A	A	A	a	a	a
12	B	A	A	A	A	H	a	a	a	a
13	LY	A	A	A	a	a	a	a	a	a

LY indicates light yellow seed coat color. B indicates the black (black and dotted) seed coat color. The alleles are abbreviated according to their origin: a, 9904 (Light yellow); A, Handel (Black); H, heterozygous. The highlighted red means the final mapping interval.

### Determination of Pigment Contents and PPO Activity

**Figure 2:**
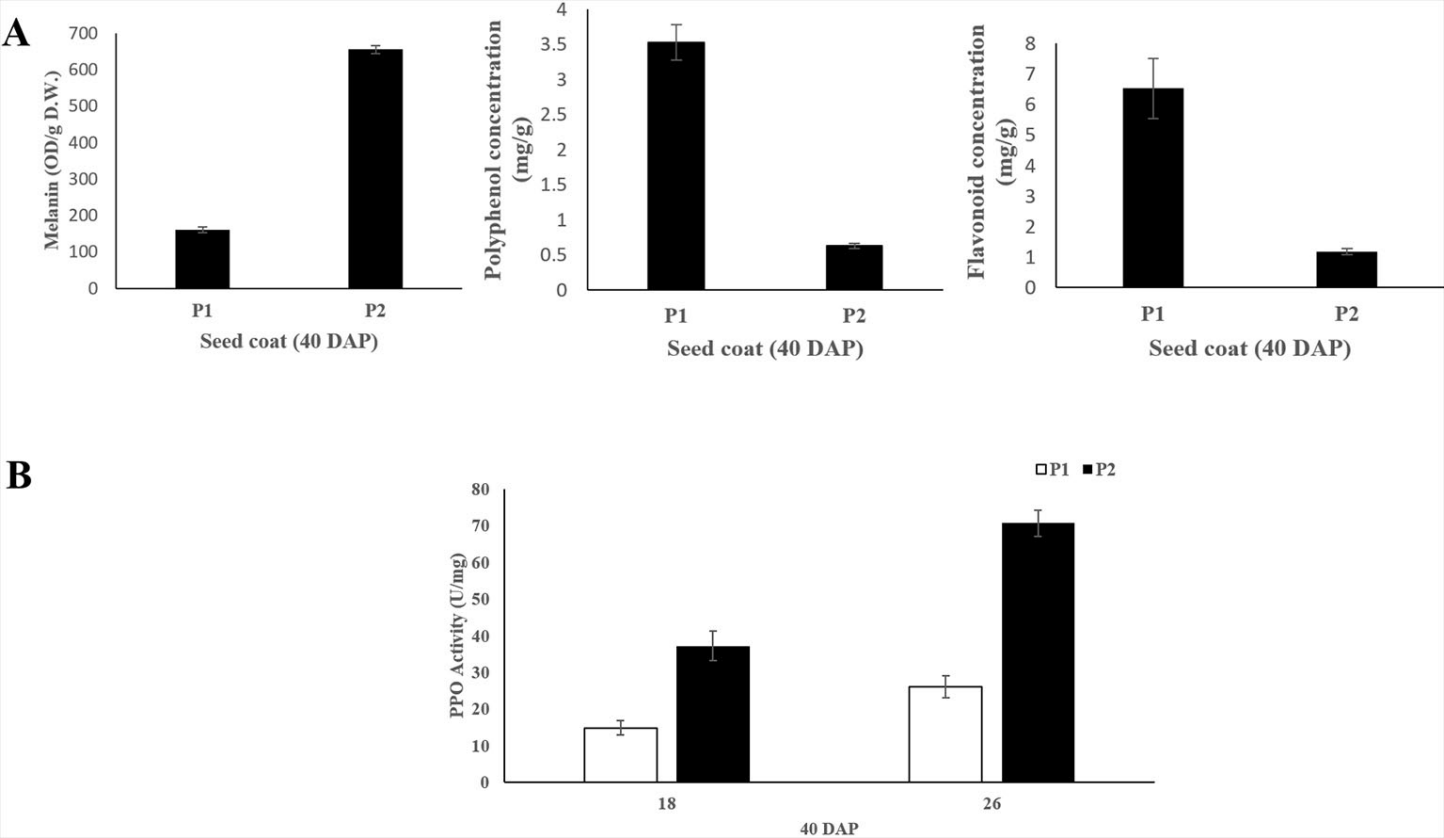
**(A)** Melanin, polyphenol and flavonoid content of seed coat at 40 DAP between the two parental lines. **(B)** PPO activity of the seed coat at 18 and 26 DAP between the two parental lines. Three biological and technical replicates were used for the measurement.

The melanin, polyphenol and flavonoid content of seed coat samples from the two parental lines at 40 DAP were measured. The results showed that black seed coat contained significantly higher (~4.4 folds) melanin content compared with light yellow seed coat (**Figure 2**). While light yellow seed coat contained higher polyphenol (~5.6 folds) and flavonoid (~8.1 folds) contents than black seed coat (**Figure 2**). PPO activity in black seed coat was 2.5 and 2.7-folds higher as compared to light yellow seed coat at 18 DAP and 26 DAP, respectively (**Figure 2**).

### Sequence and Annotation Analysis of the Candidate Genes

Based on the narrowed interval and ‘97103’ watermelon reference genome version 1 (http://cucurbitgenomics.org/), only three putative genes (*Cla019481*, *Cla019482*, *Cla019483*) were found in the 70.2 kb region (**Figure 1**). To analyze the gene sequence of the candidate genes and detect the gene responsible for seed coat color in watermelon, we designed gene specific primers to clone the whole gene and the entire coding sequence (CDS) from both parental lines (**Table S3**). The sequence alignment of the three genes between 9904 and Handel showed a base insertion existed in the CDS region of *Cla019481* at the position 106 bp in 9904 (light yellow) resulting in a frameshift mutation and leading to early termination of translation (42 residues) (**Figure 1**). A synonymous SNP mutation in the exon region of *Cla019482* leading to no amino acid change was detected. Besides, there was no sequence variation found in *Cla019483* between the two parental lines. Moreover, we have sequenced the 5’-upstream sequence from CDS region for about 2,000 bp to analyze the promoter region of the three candidate genes. There were no variations for *Cla019482* and *Cla019483* between the two parental lines, and SNPs were detected in *Cla019481* between 9904 and Handel. To verify the insertion mutation, 100 individuals were selected from the F_2_ population to check the consistency of the phenotypes and genotypes. The results indicated that 74 individuals with black seed coat (black and dotted) were homozygous dominant or heterozygous, and 26 individuals with nonblack seed coat were homozygous recessive. The phenotypes were perfectly consistent with the genotypes. Therefore, we proposed that *Cla019481* named *ClSC1* was the candidate gene for black seed coat color in watermelon.

The Cucurbit Genome Database (http://cucurbitgenomics.org/) and BLAST of the National Center for Biotechnology Information (NCBI, https://www.ncbi.nlm.nih.gov/) were used to predict the function of the candidate genes. The BLAST results indicated that all three genes in the candidate region were predicted to encode polyphenol oxidase proteins. PPO is the key enzyme to catalyze the phenolic compounds to o-quinones, which can polymerize to form the black melanin pigments attributed to black coloration during seed development (Mayer and Harel, 1979; Matheis and Whitaker, 1984; Whitaker and Lee, 1995; Yu, 2013). According to this result, *Cla019481* is considered to be the candidate gene accounting for the black seed coat in watermelon.

### Candidate Gene Expression Analysis

**Figure 3:**
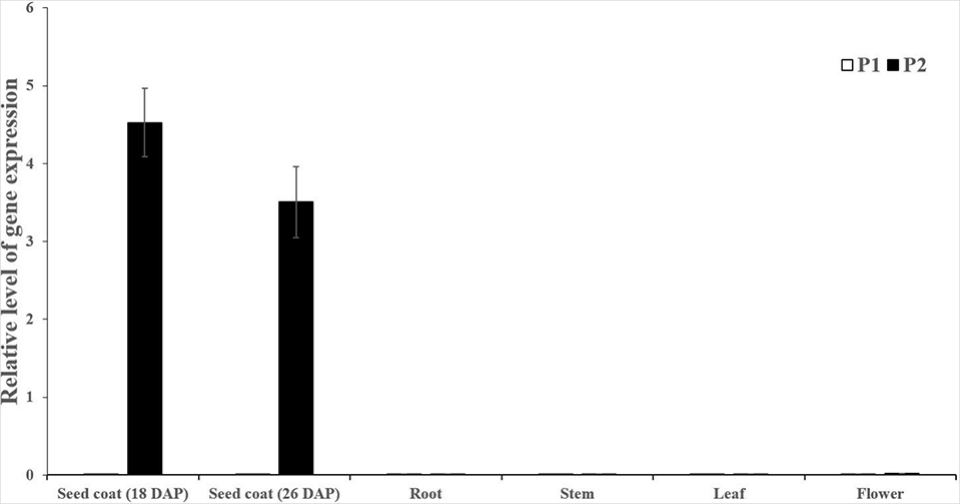
Quantitative real-time PCR analysis of *Cla019481* in different development stages (18 and 26 DAP) and different tissues. Three biological and technical replicates were used for qRT-PCR.

Seed coat samples at different development stages (18 and 26 DAP) and other tissues including roots, stems, leaves and male flowers were collected to perform expression analysis of *Cla019481* using qRT-PCR. We observed that *Cla019481* was only significantly expressed in seed coat at Handel (black seed coat; **Figure 3**). These results indicate that the different expression levels of *Cla019481* in the seed coat between the two parental lines might result in the different seed coat coloration in watermelon.

### Phylogenic and Protein Domain Analysis

To better understand the relationship between *Cla019481* protein and its homologs, we used BLAST search in NCBI database (https://www.ncbi.nlm.nih.gov/) and MEGA 7 software to perform phylogenic analysis using an alignment of the closest homologs through bootstrap method with 1,000 replications (Kumar et al., 2016). The resulting neighbor-joining tree showed that *Cla019481* from watermelon had the closest phylogenetic relationship to the homolog from *Momordica charantia* and grouped together with homologs from Cucurbitaceae family, including *Cucurbita moschata*, *Cucurbita pepo subsp. pepo*, *Luffa aegyptiaca*, *Cucumis melo*, and *Cucumis sativus* (**Figure S1**), which revealed that *Cla019481* was evolutionarily conserved within the Cucurbitaceae family.

The *Cla019481* protein domain was generated with the online Pfam database (http://pfam.xfam.org). The result showed that there were three unique domain architectures, belonging to tyrosinase, PPO1 DWL and PPO1 KFDV family, respectively (**Figure S1**). The single base insertion in CDS of 9904 resulted in a premature protein translation encoding a truncated polypeptide (42 amino acids), which is likely to be non-functional. The amino acid alignment analysis *via* NCBI and Rice Genome (http://rice.plantbiology.msu.edu/index.shtml) revealed that the *ClSC1* protein shared 44.36% sequence identity with *Phr1* (*LOC_Os04g53300*) in *Oryza sativa*, and both shared the same tyrosinase, PPO1 DWL and PPO1 KFDV domains (Yu et al., 2008).

## Discussion

The development of the high-throughput sequencing technology and the assembly of watermelon reference genome provided a powerful tool to identify loci associated with important traits (Guo et al., 2013; Wan et al., 2016). However, the marker density was far from saturated for marker-assisted selection (MAS) or for cloning important genes (Ren et al., 2015). To increase marker saturation and develop marker resources for watermelon, we constructed a high-density genetic map based on whole genome resequencing of the RIL population derived from a cross between 9904 and Handel (Li et al., 2018). We detected a prominent locus for black seed coat color in an approximate 370 kb region on chromosome 3. In the present study, we performed genetic mapping and identified a candidate gene *Cla019481* named as *ClSC1* accounting for black seed coat coloration in watermelon.

Watermelon seed coat color ranged from almost pure white to red, green, brown, tan, mahogany, and black in various superimposed complex patterns (Poole, 1941). The genetic pattern of seed coat color in watermelon is intricate and attractive. Seed coat color in watermelon was controlled by multi-genes where a certain color was dominant over others (Maina and Nyambura, 2018). According to Kanda (1931), there were at least seven genes controlling the phenotypes of seed coat color with black dominant to all colors. A three-gene model has been proposed to control the seed coat color: *r* (red), *w* (white), and *t* (tan), respectively (McKay, 1936; Poole, 1941). The interaction of these genes produced six base colors: black *(RR TT WW*); clump (*RR TT ww*); tan (*RR tt WW*); white with tan tip (*RR tt ww*); red *(rr tt WW*); and white with pink tip *(rr tt ww*) (Kanda, 1931; McKay, 1936; Poole, 1941). Furthermore, a modified gene, *d* was proposed to be responsible for a black dotted seed coat (Poole, 1941; Hawkins et al., 2001). A single pair of gene, *c_r_* was reported to account for the formation of the cracks on the seed coat (Abd el-Hafez et al., 1985). Paudel et al. (2019) found that *T* ^1^ locus was a different allele or novel locus than the previously described *T* locus and developed markers UGA3_5820134, UGA5_4591722, UGA6_7076766, and UGA8_22729513 for MAS of seed coat color in watermelon.

The phenotype of watermelon seed coat color is difficult to be classified because of the restriction in materials and various degrees of segregation. Since seed coat color is under multigenic control, advanced generations, such as recombinant inbred lines and reciprocal backcrosses, are needed to illuminate its complex inheritance and molecular mechanisms (Abdel-Hafez et al., 1985). We constructed a RIL population from a cross between inbred lines, 9904 with light yellow seeds and Handel with light yellow seeds. As compared to the other materials for seed coat color, the genetic background is relatively pure and there are only three phenotypes in the progeny (Abd el-Hafez et al., 1985; Li et al., 2018). According to the genetic analysis and previous literatures, we detected that two genes account for the seed coat color in present materials resulting in three different phenotypes, black (*W D*), dotted (*W d d*), light yellow (*w w*). The *W* allele was responsible for the black coloration, while *d* accounted for the distribution of the black in seed coat resulting in partial black named dotted. Besides, when seeds were scored as black (black and dotted) and nonblack (light yellow), the phenotype of the seed coat perfectly fit with Mendelian single gene segregation ratio, black (*W*): light yellow (*w w*), which indicated that black seed coat is controlled by a single dominant gene (*W*).

Seed coat color in watermelon is not only an important commercial trait affecting the quality especially for seeded watermelon, but also associated with water uptake, seed dormancy and germination (Mavi, 2010). Until now, few studies have been reported on QTLs or candidate genes responsible for seed coat color in watermelon and the regulatory mechanisms of seed coat coloration remains elusive. In this study, we developed a dilated F_2_ population and performed genetic mapping by CAPS and SNP molecular markers. Finally, the causal gene was delimited to a 70.2 kb region between SNP5686151 and SNP5756365 on chromosome 3. According to the watermelon reference genome, only three putative genes were annotated in this interval. The sequence alignment between the parental lines showed that one single-nucleotide base insertion existed in the CDS region of *Cla019481* at the position 106 bp in 9904 (light yellow). The SNP mutation resulted in a frameshift mutation and led to early termination of translation (42 amino acids), that likely to led to a non-functional protein (**Figure 1**). This result indicated that *Cla019481* named as *ClSC1* was the candidate gene for watermelon seed coat.

The formation and accumulation of pigments, including polyphenols, anthocyanin, flavonoid, and melanin, affect the color of seed coat (Ye et al., 2001b; Ye et al., 2001c; Zhang et al., 2006). The mechanism of seed coat coloration was well-studied in *Arabidopsis* and *Brassica* species using deficient mutants (Yu, 2013). Flavonoid (flavonols and proanthocyanidin) was responsible for the pigmentation pattern of seeds in *Arabidopsis* and *Brassica*. Dozens of genes associated with the flavonoid biosynthetic pathway have been detected using *Arabidopsis* transparent testa mutations (Yu, 2013; Appelhagen et al., 2014). Twenty-three genes have been detected at the molecular level, including enzymes (*CHS*, *CHI*, *F3H*, *F3*′*H*, *DFR*, *LDOX*, *FLS*, *ANR*, *LACCASE*), transports (*TT12*, *TT19*, *AHA10*), and regulatory factors (*TT1*, *TT2*, *TT8*, *TT16*, *TTG1*, *TTG2*, *PAP1*, *GL3*, *ANL2*, *FUSCA3*, *KAN4*) (Debeaujon et al., 2000; Baxter et al., 2005; Li et al., 2010; Yu, 2013; Padmaja et al., 2014). Due to the close phylogenetic relationship between *Arabidopsis* and *Brassica*, a number of *Brassica tt* orthologs in *Arabidopsis* have been cloned (Yu, 2013). However, compared to *Arabidopsis*, the *tt* mutants in seed coat coloration for *Brassica* do not show significant effects on morphologic and physiological performance, suggesting the presence of function complement for duplicated genes or side-pathway. There were some *Brassica* with dark brown or black seed coats indicating that the coloration in higher plants might be more complex (Yu, 2013).

In addition to flavonoids, melanin was also an important pigment for seed coat coloration, but the mechanism for seed coat coloration is still elusive and the literatures are not plentiful (Marles and Gruber, 2004; Yu, 2013). Zhang et al. (2006) reported that polyphenols, anthocyanin and flavonoid were mainly responsible for coloration in the early and middle developmental stages of the black and yellow rape-seed, but the color was mainly affected by melanin in the late stage. PPO associated with the conversion of phenolic compounds was the key enzyme in the melanin pathway (Mayer and Harel, 1979). PPO catalyzes two steps of enzymatic reactions. The first leads the ortho-hydroxylation of monophenols to ortho-diphenols. The second procedure is the oxidation of ortho-diphenols to ortho-quinone, which easily undergoes non-enzymatic reactions to form dark melanin pigments (Mayer and Harel, 1979; Matheis and Whitaker, 1984; Whitaker and Lee, 1995). PPO is the major enzyme resulting in the browned and darkened fruits, vegetables and cereals grains (Yan et al., 2017). However, the correlation between PPO and black seed coat coloration has not been well characterize. It was reported that there was significant positive correlation between PPO activity and melanin and significant negative correlation with polyphenol in *Brassica napus* seed coat (Ye et al., 2001a; Ye et al., 2002). Wei et al. (2015) proposed that *SiPPO* encoding polyphenol oxidase was responsible for generating black or whiter sesame.

PPO genes have been cloned and illustrated in different plant species, including tomato (Shahar et al., 1992), potato (Hunt et al., 1993), grape (Dry and Robinson, 1994), apple (Boss et al., 1995), wheat (Demeke and Morris, 2002), rice (Yu et al., 2008), barley (Taketa et al., 2010), broad bean (Taketa et al., 2010). However, no PPO-like genes were identified in *Arabidopsis* and chlorophyte (green algae) (Tran et al., 2012). All of the PPO genes have two conserved copper-binding domains (CuA and CuB), forming the central domain of the catalytic site (He et al., 2007). In rice, *Phr1* encoding PPO contributed the discoloration of hulls and coarse grains of *indica*-type cultivars, while the nonfunctional *Phr1* leaded to no discoloration of *japonica* grains (Yu et al., 2008). The insertion or deletion resulting in frameshift mutations in the *Phr1* CDS accounted for the PHR (phenol reaction)-negative phenotype in 35 japonica lines (Yu et al., 2008). According to watermelon reference genome, there are eight PPO genes and three of the genes are found in the present narrowed region. Based on the sequence analysis, *ClSC1* encoding polyphenol oxidase enzymes was the casual gene for the black seed coat coloration.

Black seed is attractive with scarlet or canary yellow flesh and is considered as a promising source of the natural melanin, which shows remarkable activities of antioxidant, radio-protective, thermoregulative, antitumor, immunostimulating and anti-inflammatory (Yan et al., 1996; Łopusiewicz, 2018). In the present study, significantly higher melanin content was found in black seed coat compared with light yellow seed coat. Besides, polyphenols content in light yellow seed coat was much higher than in black seed coat (**Figure 2**), which is in harmony with *B. napus* (Ye et al., 2002). For this phenomenon, we hypothesized that a large amount of polyphenols in black seed coat were used to synthesize melanin through the catalyzation of PPO, resulting in the pigment difference in black and yellow light seed coat (Ye et al., 2002). In this study, sequence alignment between light yellow seed coat and black seed coat showed that a single-nucleotide (A) insertion within the *ClSC1* in 9904 (light yellow) resulted in a frameshift mutation and premature stop codon (**Figure 1**). The mutated *ClSC1* encoded a non-functional truncated protein (42 amino acid). In addition, *ClSC1* specifically showed significantly high level of transcript in Handel with black seed coat (**Figure 3**). Moreover, there was significantly higher PPO activity in black seed coat compared with light yellow seed coat (**Figure 2**). Hence, it is hypothesized that the different expression of *ClSC1* accounts for the different activities of PPO in the seed coat, which resulted in the different amount of melanin forming the black and light yellow seed coat.

In general, we illustrated the inheritance pattern of seed coat color and suggested a causal gene *ClSC1* accounting for the black seed coat coloration in watermelon. Our results further facilitate marker-assisted breeding and provide further evidence to understand the molecular mechanisms in seed coat coloration in watermelon and other crops.

## Data Availability Statement

All datasets generated for this study are included in the article/**Supplementary Material**.

## Author Contributions

WL and XL conceived the research and designed the experiments. NH developed the plants population. SZ, PY and CG analyzed data. HG and UM checked the manuscript. BL performed most of the experiment and wrote the manuscript. All authors reviewed and approved this submission.

## Funding

This research was supported by the Agricultural Science and Technology Innovation Program (CAAS-ASTIP-2016-ZFRI-07), National Key R&D Program of China (2018YFD0100704), the China Agriculture Research System (CARS-25-03) and the National Nature Science Foundation of China (31672178 and 31471893).

## Conflict of Interest

The authors declare that the research was conducted in the absence of any commercial or financial relationships that could be construed as a potential conflict of interest.

## Supplementary Material

Supplementary Figure 1 The analysis of the phylogenetic and conserved domains of the candidate gene. **(A)** The phylogenetic tree of Cla019481 and its homologous proteins. **(B)** The conserved domains were analyzed by online Pfam database.

Supplementary Table 1 The segregation ratio of seed coat color among different populations.

Supplementary Table 2 The information of CAPS and SNP markers on chromosome 3 used for the polymorphic analysis.

Supplementary Table 3 The primers information used for the sequence and expression analysis of the candidate genes.
