# Supplementary figures and images for: Genetic Mapping and Discovery of the Candidate Gene for Black Seed Coat Color in Watermelon (Citrullus lanatus)
Source: Front Plant Sci. 2020 Jan 22;10:1689. doi: 10.3389/fpls.2019.01689 (PMC6987421; doi:10.3389/fpls.2019.01689)

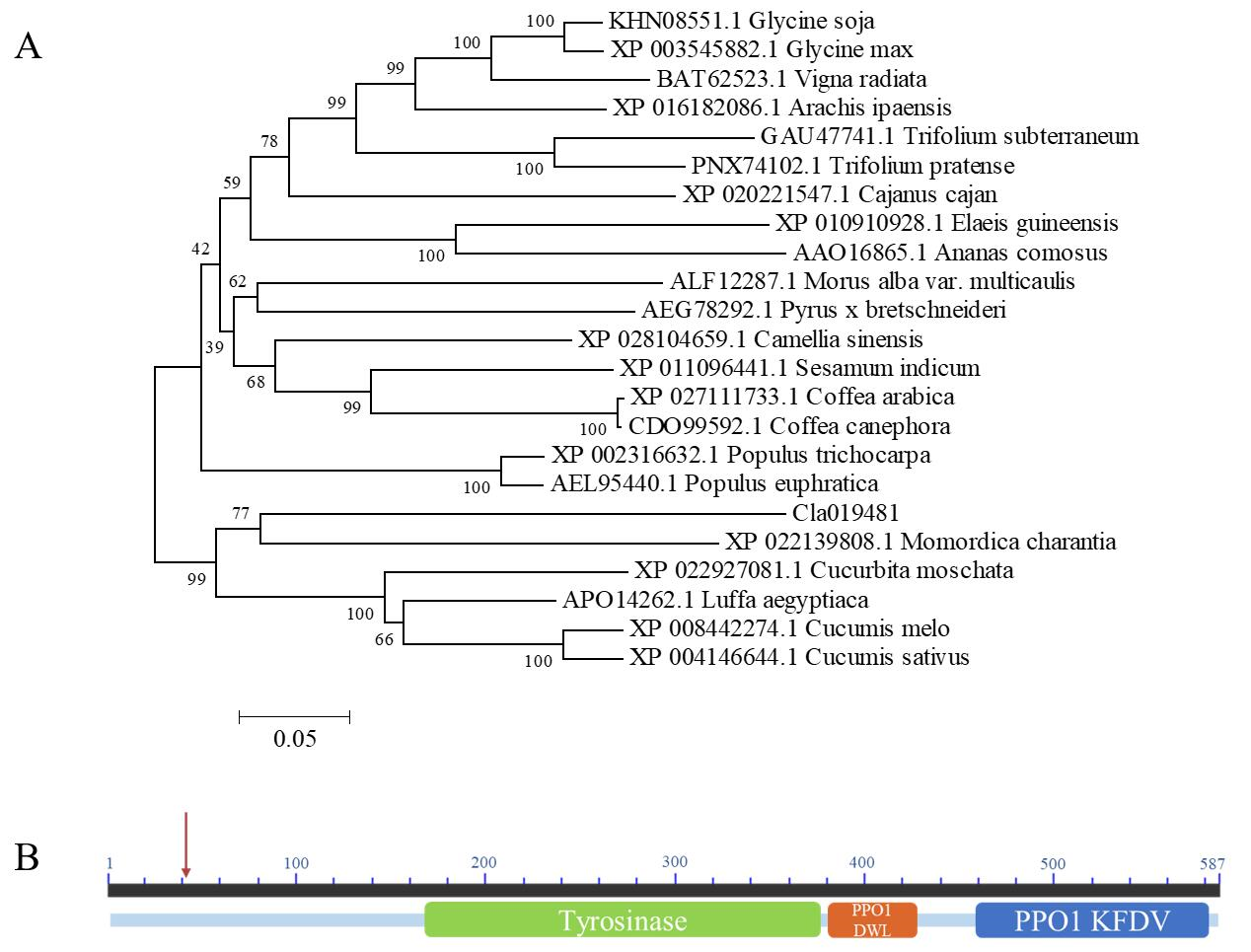

Supplement: Supplementary Figure 1 — The analysis of the phylogenetic and conserved domains of the candidate gene. (A) The phylogenetic tree of Cla019481 and its homologous proteins. (B) The conserved domains were analyzed by online Pfam database. [file Image_1.tif]
